# Cellular and Molecular Mechanisms Underlying Tricuspid Valve Development and Disease

**DOI:** 10.3390/jcm12103454

**Published:** 2023-05-14

**Authors:** Nadia Salerno, Giuseppe Panuccio, Jolanda Sabatino, Isabella Leo, Michele Torella, Sabato Sorrentino, Salvatore De Rosa, Daniele Torella

**Affiliations:** 1 Department of Experimental and Clinical Medicine, Magna Graecia University, 88100 Catanzaro, Italy; nadia.salerno@unicz.it (N.S.); i.leo@unicz.it (I.L.); 2 Department of Medical and Surgical Sciences, Magna Graecia University, 88100 Catanzaro, Italy; panuccio@unicz.it (G.P.);; 3 Department of Translational Medical Sciences, University of Campania Luigi Vanvitelli, 80138 Naples, Italy

**Keywords:** tricuspid valve, embryology, cellular mechanisms, molecular mechanisms, regeneration

## Abstract

Tricuspid valve (TV) disease is highly prevalent in the general population. For ages considered “the forgotten valve” because of the predominant interest in left-side valve disease, the TV has now received significant attention in recent years, with significant improvement both in diagnosis and in management of tricuspid disease. TV is characterized by complex anatomy, physiology, and pathophysiology, in which the right ventricle plays a fundamental role. Comprehensive knowledge of molecular and cellular mechanisms underlying TV development, TV disease, and tricuspid regurgitation-related right-ventricle cardiomyopathy is necessary to enhance TV disease understanding to improve the ability to risk stratify TR patients, while also predicting valve dysfunction and/or response to tricuspid regurgitation treatment. Scientific efforts are still needed to eventually decipher the complete picture describing the etiopathogenesis of TV and TV-associated cardiomyopathy, and future advances to this aim may be achieved by combining emerging diagnostic imaging modalities with molecular and cellular studies. Overall, basic science studies could help to streamline a new coherent hypothesis underlying both the development of TV during embryogenesis and TV-associated disease and its complications in adult life, providing the conceptual basis for the ultimate and innovative field of valve repair and regeneration using tissue-engineered heart valves.

## 1. Introduction

For ages considered “the forgotten valve” because of the predominant interest in left-side valve disease, the tricuspid valve (TV) has now received significant attention in recent years. Whereas tricuspid stenosis is very rare, tricuspid regurgitation (TR) is frequently diagnosed, affecting up to 65–85% of the general population. In most cases, TR is secondary to adverse remodeling of the right ventricle (functional TR) caused by left heart disease (62.4% of all TR) or pulmonary hypertension. Primary/organic TR and isolated TR represent about 4.8% and 8.1% respectively, while only in a small percentage, about 1.7%, TR is congenital. According to the aging population, TR prevalence is expected to increase, similarly to other valve diseases [1,2]. In the community setting, all-cause TR is more frequent in women than in men, although the mechanisms underlying this sex difference are still unknown [3]. More relevantly, despite previously being considered well-tolerated, recent evidence shows that significant TR, even when isolated, is associated with high mortality rates [4].

Although it has high prevalence and poor outcomes, TR remains an untreated disease when compared to left-side valve diseases. In most cases, optimal medical therapy is the only treatment offered. The advance in imaging and intervention techniques have significantly evolved the assessment and management of TV disease during the last years. With recent advances in transcatheter valve intervention, the anatomy and embryology studies of the TV have received renewed attention [5]. Moreover, due to the limitations of prostheses currently available, several attempts have been made to develop new devices since the 1900s, opening the field of heart valve tissue engineering [6]. The main purpose of this review article is, therefore, to critically review the available novel basic science findings assessing TV disease and TV-associated cardiomyopathy. Studies of TV development are illuminating the path forward and suggest unique opportunities for heart regeneration, particularly in fetal and neonatal periods. Here, we also review major lessons from heart valve development that inform current and future studies directed at enhancing TV disease’s understanding and effective therapeutic strategy. Overall, this evidence of basic science studies could help to streamline a new coherent hypothesis underlying both the development of TV during embryogenesis and TV-associated disease and its complications in adult life, providing the conceptual basis for the latest evidence in the innovative field of valve repair and regeneration.

## 2. Tricuspid Valve Development: Embryology and Anatomy

The TV is the atrioventricular valve anatomically connected to the morphological right ventricle. In normal hearts, it is closer to the apex, anteriorly, and to the right of the mitral valve (MV). The effective functioning of the TV depends on the structural and functional integrity of each component of the “tricuspid valve–atrioventricular complex”, which includes the leaflets, chordae tendineae, papillary muscles, tricuspid annulus (TA), right atrium (RA), and right ventricle (RV) [7]. Compared to the MV, the three TV leaflets are thinner, more translucid, and flimsy. They differ in size and shape, with the anterior leaflet (AL) being the largest, the most robust, and having a quadrangular shape, the posterior leaflet (PL) being triangular, and the septal leaflet (SL) being the most fixed and semicircular, with scalloped indentations and chordae originating directly from the septum. Of note, in healthy subjects, the number of leaflets is variable, and recently a simplified nomenclature has been proposed [8]. The papillary muscle variability is another distinctive characteristic of the TV; they are smaller and more numerous compared with those of the left ventricle. Two to nine papillary muscles can be enumerated in each different RV, but they are usually two or three: the anterior, the posterior, and the septal papillary muscle. The anterior papillary muscle is typically the most prominent, located near the apex on the anterior wall of the RV, and merging with the moderator band. The posterior papillary muscle may be bifid or trifid. The septal papillary muscle, which is diminutive and frequently absent, has multiple chordal attachments originating directly from the ventricular wall [9]. The TA is so tiny that is difficult to identify as a true “fibrous ring” when compared to the mitral valve, whereby annular calcifications are almost nonexistent during a lifetime. It is a saddle-shaped or elliptical non-planar structure with the medio-lateral diameter being larger than the antero-posterior diameter. However, the TA is a highly dynamic structure that changes in shape and size during respiratory and cardiac cycles, due to the surrounding myocardium’s contraction [10]. In chronic cardiac pathologies, such as long-standing TR, the TA becomes a planar structure. From a functional standpoint, the TV serves to impede the reverse flow of blood from the RV into the RA. Importantly, even the cyclical oscillation of caval flow is generated by the rhythmic opening and closing of the TV, which occurs during ventricular diastole and ventricular systole, respectively.

Three anatomical structures close to the TA are at risk of injury during interventional procedures: the non-coronary sinus of Valsalva, the bundle of His, which runs beneath the membranous septum, and the right coronary artery, which runs down the right atrioventricular (AV) groove and anterior to the anterior TV leaflet. The triangle of Koch, which is located in proximity to the TV, is of particular importance when studying the conduction system. This triangle is defined by three anatomical structures: the coronary sinus ostium at its base, the tendon of Todaro, and the septal leaflet of the TV. The apex of this triangle contains the atrioventricular (AV) node, the central fibrous body penetrated by the His bundle, and the membranous septum lying anteriorly. These structures are in such proximity to the AV node that they are susceptible to damage during surgery [11]. Moreover, diverse congenital conditions of the TV are associated with an arrhythmogenic substrate [12,13].

The TV complex originates from the endothelial cells located in the endocardial AV cushion tissue, which divides the atria and ventricles and assists in creating the AV septum. The septal leaflet primarily emerges from the inferior endocardial cushion, with a minor contribution from the superior cushion. Subsequently, the leaflet delaminates from the myocardium. The anterior and posterior leaflets arise from the invagination of a segment of the ventricular myocardium, comprising the lateral mesenchymal cushion, subepicardial mesenchyme, and myocardium of the AV groove. This process of tunneling inward commences at the lower part and progresses upward until it reaches the AV junction. Then, the adjacent muscle tissue undergoes controlled disintegration, ultimately producing the valvular leaflets. Additionally, the regressive ventricular wall tissue also plays a role in the formation of the chordae tendineae [14].

## 3. Developmental Mechanisms Implicated in Tricuspid Valve Disease

**Figure 1:**
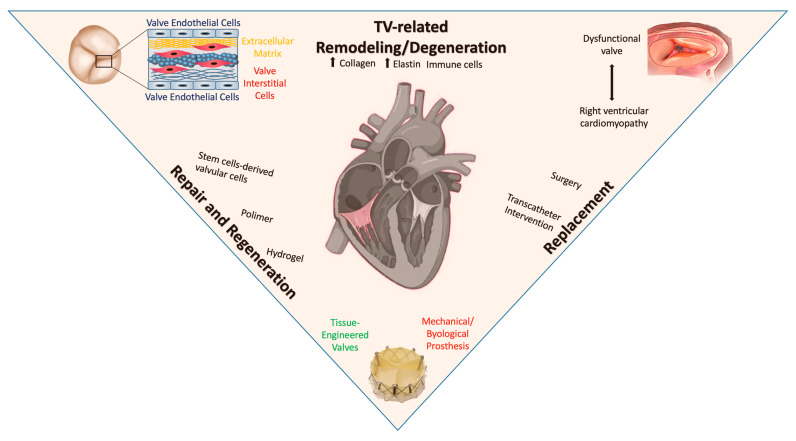
The cartoon depicts the main cellular events underlying tricuspid valve (TV) disease, and TV remodeling and degeneration in particular, leading to right ventricular (RV) cardiomyopathy and TV dysfunction. These molecular and cellular aspects set the basis for innovative strategies for valve repair and regeneration using novel biomaterials and/or stem cell products for tissue-engineered biological valves, for surgical or transcatheter TV replacement.

Dysfunction or failure of some of the molecular and morphogenetic mechanisms underlying tricuspid and right structures development have been proven to be associated with tricuspid disease (Figure 1). Ebstein’s anomaly (EA) is a rare condition accounting for <1% of all congenital heart diseases (CHDs), and is characterized by an apical displacement of the posterior and septal TV leaflets [15]. The hinge point of the anterior leaflet is usually retained in a normal position, although the leaflet itself is often elongated and tethered, with fenestrations and a ‘sail-like’ appearance [15,16]. The combination of the described features causes displacement and geometrical distortion of the valvular orifice, resulting in various degrees of tricuspid regurgitation and/or stenosis, enlargement of the RA, and enlargement of the “atrialized” portion of the RV, which is thinner and poorly contractile. The degree of apical leaflets displacement and the consequent extent of RV “atrialized” myocardium has a direct consequence on the RV functional capacity, and influences clinical outcomes [17]. Recent studies have shown that abnormal developmental mechanisms, particularly failure in the separation of the inner layer of the endocardium from the ventricular wall (a process known as “delamination”), can contribute to EA development [18]. The leaflets remain adherent to the underlying myocardium with a consequent displacement towards the apex and a rotational shift that also involves the valvular apparatus. The bone morphogenetic proteins (BMPs) signaling pathway has a key role in the differentiation of endocardial cushion mesenchymal cells; mutations in genes involved in this pathway have been found in a genotyping study involving 47 isolated EA cases and have been associated with ventricular preexcitation, often accompanying EA [19,20]. However, this is not the only genetic basis identified as potentially responsible for the disease. Mutations in NK2 homeobox 5 (NKX2-5) and GATA binding protein 4 (GATA4), transcription factors regulating cardiac development and AV valve formation, have been also described in EA [15,21]. In addition, mutations in the sarcomeric gene Myosin Heavy Chain-7 (MYC-7) have been described in EA patients. Interestingly, the same gene is often mutated in patients with left ventricular non-compaction, and cases with overlapping conditions have been described in the literature [22,23]. Several environmental factors can interfere with the delamination process, although the exact mechanism has not been yet fully understood. Hypoxia and maternal exposure to lithium and benzodiazepine have been all associated with an increased risk of EA [24,25]. Incomplete failure of the delamination process may instead result in tricuspid dysplasia, a rare congenital abnormality sharing common features with EA, but characterized by a preserved hinge point of the valvular leaflets [26]. The leaflets may be thickened and elongated with variable degrees of associated regurgitation. Papillary muscles and cordae are also frequently abnormal, shortened, or even absent [27]. However, the absence of the “atrialized” RV guarantees preserved myocardial contractility, and the associated RV cardiomyopathy is rarer compared to EA. In EA, AV reciprocating tachycardias (AVRTs) are the most common finding, and approximately 25% of cases are associated with Wolff-Parkinson-White syndrome (WPW). The downward displacement of the septal TV leaflet is associated with discontinuity of the central fibrous body and septal AV ring with direct muscular connections, which creates a potential substrate for accessory AV connections and ventricular pre-excitation. Multiple accessory pathways (APs) are often located around the orifice of the malformed TV, especially in the region of the septal and posterior (mural) leaflets. Occasional sudden death in patients with Ebstein’s anomaly is thought to be due to atrial fibrillation (AF) in the presence of WPW syndrome [12]. Re-entry is possible even around the atretic TV in patients with tricuspid atresia, in whom both congenital and surgically acquired accessory pathways might be accountable for the heightened incidence of WPW [13].

Tricuspid atresia (TA), characterized by the complete absence of the TV, is the third-most common cyanotic congenital condition, accounting for 1–3% of congenital heart diseases [28]. The concomitant presence of an atrial septal defect (ASD) and ventricular septal defect (VSD) allows communication between pulmonary and systemic circulation. Although the underlying mechanisms leading to congenital TV atresia are not yet fully established, alterations in the GATA-4 signaling pathway also seem to have a key role in this defect [29,30]. Friend of GATA-2 (Fog2) is a multi-zinc-finger protein co-expressed with GATA-4 during cardiac development [31]. It has been demonstrated that FOG-2-deficient mice develop tricuspid atresia with associated ASD and VSD, suggesting a genetic basis for this syndrome [30].

Atrioventricular canal defect (AVCD) has an estimated prevalence of approximately 5% among congenital malformations [32]. The classification of AVCD includes the complete form, with a common AV valve associated with both an ostium primum ASD and a VSD, the partial AVCD, with two distinct AV valve orifices (often associated with a cleft of the anteromedial leaflet of the left-sided valve), and the intermediate AVCD (partial AVCD plus a restrictive VSD) [33]. Failure in the development of the endocardial cushion, with completely absent or only partially fused AV cushions, has been traditionally considered the primary mechanism underlying the disease [33]. Confirming this hypothesis, mutations in GATA4 have been associated with AVCD spectrum disease [34].

More recently, attention has been paid to additional mechanisms that may play a role in the development of AVCD. AV septation requires the fusion of three structures: the endocardial cushions (superior and inferior), the mesenchymal cap (a cushion-like structure located at the edge of the primary atrial septum), and the dorsal mesenchymal protrusion (DMP) [18,35]. The latter, also known as “spina vestibuli”, derives from the second heart field (SHF) and forms the inferior muscular portion of the atrial septum [36]. Developmental abnormalities of DMP have been associated with AVCD, revolutionizing the “endocardial cushion defect” paradigm. Anomalies in bone morphogenetic protein (BPM) [19,37], Tbx5 [38], NKX2.5 [37,39], CRELD1 [37,40], Wnt/β-Catenin [41] and Sonic Hedgehog [42] signaling pathway, all implicated in DMP formation, have been found in association with AVCD.

The development of the heart originates with the formation of a heart tube, consisting of mesodermal progenitors fused along the midline. This primitive heart tube will elongate during embryonic development. Its most cranial part, looping to the right, will form the future RV, whilst the middle part, displaced to the left during the looping process, will constitute the future LV. Complete transposition of great arteries (d-TGA) refers to a congenital anomaly in which the rightward loop is preserved and the morphologically RV is at the right of the LV. The aorta will, therefore, originate from the RV (rightly and anteriorly displaced) and the pulmonary artery from the LV (ventriculo-arterial discordance). The two great vessels will run in parallel, with no communication between systemic and pulmonic circulation. Other lesions are therefore commonly associated, maintaining a communication between the two systems. In these patients, a degree of tricuspid regurgitation is also very common due to abnormalities of the valvular apparatus, iatrogenic damage during previous surgery, or annular dilatation. In the congenitally corrected transposition of the great vessels (CCTGA), also known as L-TGA, the heart tube is instead looped leftward, with the morphological RV located on the left of the morphological LV. A double discordant atrioventricular and ventriculo-arterial connection is the hallmark of the disease, with various clinical courses ranging from early symptomatic patients to patients who remained undiagnosed until late adulthood [43]. Abnormalities of the systemic (tricuspid) AV valve will be found in up to 90% of these patients, often mimicking EA with some substantial differences. The adhesion of the septal and posterior leaflets to the myocardium is, in this case, very limited, and the “sail-like” appearance of the anterior leaflet may instead present a cleft or interfere with the outflow.

The systemic right ventricle (SRV) encounters a series of adaptive mechanisms needed to support systemic circulation, eventually leading to SRV dilatation, hypertrophy, and systolic dysfunction [43]. The overall increased arrhythmic burden and the concomitant TR, due to both annular dilatation and a various degree of valve apical displacement mimicking EA, further contribute to SRV failure [43]. Another critical step during heart development is the “wedging” process that, through a counterclockwise rotation of the outflow tract, allows a correct alignment of the aorta behind the pulmonary artery and the final positioning of the aortic valve between the atrioventricular valves. SHF cells and cardiac neural crests cells are implied in outflow tract formation and great vessels septation [44,45]. Failed migration of these progenitor cells during the wedging process is one of the mechanisms implied in the conotruncal abnormalities including, among others, TGA. From a molecular point of view, alterations in TBX1 expression and Nodal and Notch signaling implicated in the regulation of cardiac cell fate and left-right asymmetry have all been associated with the defect [46,47,48,49].

## 4. Postnatal Valve Remodeling

The complex biomechanical characteristics of the cardiac valves enable them to work in directed blood flow throughout the cardiac cycle. Extracellular matrix (ECM), valvular interstitial cells (VICs), and overlying endothelial cells (EC) make up the leaflets of the mature AV valves, which include MV and TV [50,51]. The highly organized and compartmentalized ECM composition of the leaflets is primarily responsible for meeting the mechanical requirements of the valve for elasticity, compressibility, stiffness, and strength, as well as durability throughout an individual’s lifespan. Although the development of heart valves in the embryo is well understood, postnatal valve ECM remodeling’s regulatory mechanisms are less clear. Moreover, postnatal VICs in healthy individuals change transcriptionally from being slightly proliferative to becoming steadily more quiescent [52]. Mature heart valves are made up of VICs and cells with hematopoietic origins that are embedded in a stratified ECM and encircled by valve endothelial cells (VEC). Heart valves are not fully developed at birth, and postnatal ECM remodeling in humans and mice results in different ECM layers. The postnatal remodeling of the TV begins immediately after birth and has two crucial moments: postnatal day 7 (P7) and postnatal day 30 (P30). By P30 in mice and during childhood in humans, heart valve remodeling includes elongation of valve leaflets with increased collagen and elastin production that results in the formation of collagen-, proteoglycan-, and elastin-rich layers. Contrary to P1, when collagen expression is low, valve leaflets begin to form a collagen layer at P7; however, the primitive leaflets continue to be thick. At P30, the valve leaflets are elongated, and fibrillar collagen and proteoglycan are distributed regionally [53]. Transcriptomic single-cell RNA sequencing analyses showed that P7 cells contain five cell clusters, while P30 cells contain seven cell clusters. This cell clustering distinguishes immune, endothelial, interstitial, and melanocyte subpopulations of cells. In more detail, analyses of developing heart valves reveal that the immune cells present in heart valves are made up of T cells, mast cells, dendritic cells, and macrophages. In addition, in the P7 and P30 heart valve leaflets, three endothelial cell populations with distinct spatial locations have been found. Concerning VICs, P7 VICs have increased synthetic activity and enhanced biological functions related to the structuring of collagen fibrils, peptide crosslinking, and bone formation. They can be divided into two subsets, one producing collagen α1 (almost 50% of VICs population) and another devoted to the production of glycosaminoglycan (GAG). At P30, VICs are divided into four subsets, different from P7 and distinguished in matri-fibrocytes, fibrosa-VICs, Tcf21 VICs (expressing genes involved in defense response), and antigen-presenting cells. Those cells express complement factors, ECM proteins, and osteogenic genes differently and are leaflet-specific [54]. Therefore, while interstitial cell subpopulations change in gene expression and cellular functions in primordial and mature valves, endothelial and immune cell subpopulations remain largely constant throughout postnatal development. The role of melanocytes in TV is still debated. Those cells derive from neural-crest-derived cells, but their function in TV is still unclear [55,56]. Their timing and arrival in the heart imply that they may have a role in the formation of the AV valve from the endocardial cushions. The expression of certain ECM proteins and remodeling enzymes such as metalloproteases are necessary for this process [57]. Melanocyte precursors may aid in the remodeling of the valve because they express and produce metalloproteases [58]. This role may persist slowly into maturity, when melanocytes and interstitial cells may contribute to the preservation of tissue homeostasis and influence the mechanical characteristics of the AV valves. Therefore, one of the hypotheses related to melanocyte’s role in TV is that they could influence ECM. While there are no keratocytes in the heart, melanin produced by melanocytes could form melanin-protein aggregates which increase stiffness in comparison to TV non-pigmented areas [59,60].

## 5. Right Ventricular Cardiomyopathy Induced by Tricuspid Valve Disease

**Figure 2:**
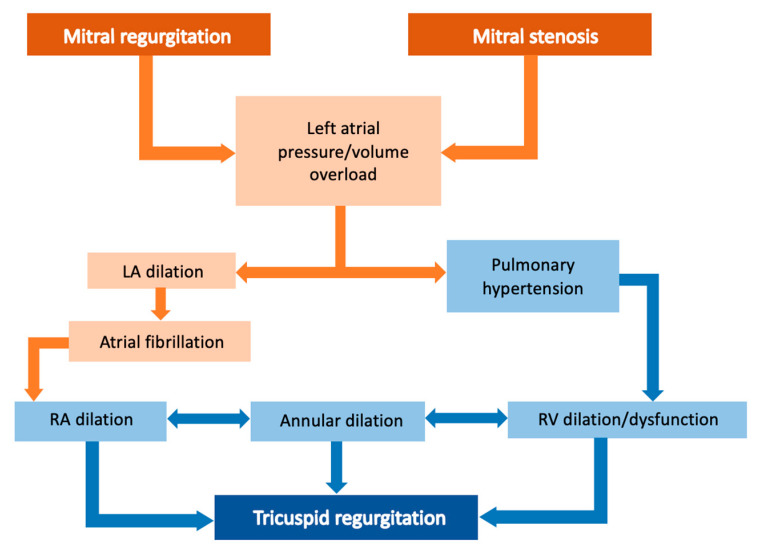
Pathogenetic mechanisms of tricuspid regurgitation in mitral valve disease.

It has long been known that TR concomitant to left-sided cardiac valve dysfunction increases the risk of early and late mortality following surgery [61,62]. Over the years, TR has been considered a simple functional consequence of left-sided heart disease due to RV and consequently TA dilatation [63]. However, RV cardiomyopathy and TR are not only functionally related to left-sided valve diseases. Between 25% and 40% of individuals with severe mitral regurgitation (MR) or aortic stenosis (AS), respectively, have TR [64]. The pathogenesis of TR in mitral valve disease is complex and multifactorial (Figure 2). Mitral valve disease results in an increase of left atrial pressure, which in turn can cause secondary pulmonary hypertension. Pulmonary hypertension may further lead to RV dysfunction and remodeling, TA dilation, displacement of papillary muscles, and tethering of the TV leaflets. Additionally, the left atrial enlargement may lead to the development of atrial fibrillation. This, in turn, can cause dilation of the right atrium and further TA dilation.

Nevertheless, MR or AS surgical therapy is typically not followed by an improvement in TR; it may, indeed, be followed by a progression or even a late development of TR, showing that TR is not only a consequence of the hemodynamic state induced by left-sided heart valves disease [65]. Right ventricular cardiomyopathy induced by TR is various. Functional TR is secondary to the dilatation of the TA, which is linked to RV dilatation. As shown in anatomic and 3-Dimensional studies, TA dilatation moves anteroposteriorly along the RV-free wall, because the septal section of the TA is more fibrous and attached to the interventricular septum [66]. In functional TR, with a smaller mediolateral to anteroposterior ratio, the TA loses its typical saddle shape and elliptic form, becoming flatter and more circular in contrast to healthy control patients. The tricuspid area grows and TA excursion decreases. The interaction between the papillary muscle and the leaflet and the TA may change because of the flattening of the TA that takes place with TR. The low points of the annulus may be stretched away from the papillary muscles when the annulus flattens, causing tethering. Functional TR can also emerge from changes in RV geometry that are not caused by TA dilatation, such as papillary muscle dislocation secondary to RV dilatation, resulting in tethering of the TV leaflets and consequent TR [67].

RV cardiomyopathy induced by TR has two main mechanisms: RV dilatation and RV dysfunction. Both components have a significant impact on the prognosis of patients with TR [68]. Significant TR causes volume overload, which causes the RV to progressively enlarge and become dysfunctional. RV remodeling has not been well examined, thus it is unclear if RV dilatation and dysfunction always coexist. Dilation and dysfunction, the two components of RV remodeling, could each have a distinct effect on prognosis. Therefore, RV remodeling can be characterized into four patterns: (1) normal RV size and function; (2) RV dilatation without RV dysfunction; (3) normal RV size with RV dysfunction; (4) RV dilatation with RV dysfunction. Different forms of RV remodeling can also affect the prognosis of patients with severe TR. As a matter of fact, in 1292 patients with severe functional TR, the presence of RV remodeling patterns 3 and 4 was related to a lower five-year survival rate as opposed to pattern 1 RV remodeling (52% and 42% vs. 70%; *p* = 0.002 and *p* < 0.001) [69].

Mechanisms underlying TR are strongly related to the RV remodeling pattern that may occur in the development of this valve disease, demonstrating the wide variety of this phenomenon. Significant secondary TR may appear to be caused by right atrial enlargement and atrial fibrillation in individuals without left-sided cardiac disease or pulmonary hypertension (so-called isolated TR), while the RV’s size and function are within normal limits. As shown by Mutlak et al. [70], among 242 patients with severe TR, 23 patients (9.5%) were found to have significant TR without significant pulmonary hypertension or left-sided heart disease. RV enlargement was observed in 47% of these patients, and the typical findings included TA dilation and atrial fibrillation. According to Topilsky et al. [71], pulmonary hypertension-related TR was linked to increased RV length (elliptical deformation), which resulted in tenting of the tricuspid leaflets, whereas idiopathic TR was linked to basal RV enlargement (conical deformation) and TA dilatation.

In patients with left-sided cardiac disease and pulmonary hypertension, there are various grades of RV dilatation and dysfunction. Therefore, advanced RV remodeling is more frequent in patients with severe MR, AS, or severe left ventricle (LV) dysfunction. RV pressure overload results from these disorders’ normal course of progressive LV remodeling with LV hypertrophy, dilatation, and higher LV filling pressures that pass to the left atrium and pulmonary circulation. To enhance RV preload and be able to elevate mean pulmonary arterial pressure to >60 mm Hg, the thin-walled RV reacts with myocardial hypertrophy and dilatation, in order to preserve RV systolic performance [72]. The tricuspid valve annulus may dilate because of this remodeling process, and the tricuspid valve leaflets may become tethered, creating considerable TR and volume overload that raises RV dimensions and wall stress. RV coronary blood flow and contractility could be impaired if chronically elevated afterload (pressure overload) and preload (volume overload) are not managed. Furthermore, myocyte replacement and loss as well as myocardial fibrosis may take place, which lowers the likelihood of RV functional recovery following TR correction and affects survival. To properly plan the time of tricuspid valve intervention, it is important to characterize RV remodeling in patients with substantial TR.

TR-induced right ventricular cardiomyopathy has a molecular and cellular basis which is still not completely clear. RV cardiomyopathy is a complex phenomenon, in which long non-coding RNAs (lncRNAs) and immune infiltration have been identified to have a crucial role in its occurrence and development. In more detail, 648 differently expressed mRNAs, 201 differentially expressed miRNAs, and 163 differentially expressed lncRNAs were found in nine cases of TR-induced RV cardiomyopathy in comparison with nine controls with normal RV by Tian et al. [73]. In this study, the hub genes ADRA1A, AVPR1B, OPN4, IL-1B, IL-1A, CXCL4, ADCY2, CXCL12, GNB4, CCL20, CXCL8, and CXCL1 have been identified by protein–protein interaction network analysis. CTD-2314B22.3, hsamiR-653-5p, and KIF17ceRNA; SRGAP3-AS2, hsa-miR-539-5p, and SHANK1; CERS6AS1, hsa-miR-497-5p, and OPN4; INTS6-AS1, hsa-miR-4262, and NEURL1B; TTN-AS1, hsa-miR-376b-3p, and TRPM5; and DLX6-AS1, hsa-miR-346, and BIRC7 axes were obtained by constructing the competing endogenous RNA (ceRNA) networks. In addition, the examination of immune infiltration showed that in TR-induced RV cardiomyopathy, the percentage of CD4 and CD8 T cells was around 20%, and the percentage of fibroblasts and endothelial cells was significant.

## 6. Valvular Repair and Regeneration

For over 50 years, TR has been considered a minor issue, and patients are often referred for treatment very late, when they have developed significant RV enlargement or dysfunction. However, recent evidence has shown that significant TR can have negative effects on long-term clinical outcomes. For many years, the preferred treatment for dysfunctional heart valves has been surgical repair or replacement using mechanical or bioprosthetic valves. Specifically, tricuspid annuloplasty is the preferred surgical therapy for functional tricuspid regurgitation as it corrects TA dilatation and restores annular geometry. When TV repair is not possible or not likely to be successful, TV replacement is the alternative treatment. In recent years, the development of transcatheter interventions has revolutionized the treatment approach of valve disease and has become a viable alternative to surgery, especially for high-risk patients [74]. Different approaches include coaptation devices, annuloplasty devices, caval valve implantation (CAVI), and transcatheter tricuspid valve replacement (Figure 1). These advances have greatly improved patients’ survival rates and quality of life.

Despite these continuous improvements, an ideal heart valve prosthesis that satisfies all the necessary functional and anatomical properties has not yet been developed. Such a prosthesis would need to be adaptable to functional changes, while being inert and resistant to infections and thrombogenicity [75] and possess a self-growth and self-renewal capacity. Tissue engineering has been attempting to overcome these limitations since 1993, when Langer and Vacanti introduced this new paradigm based on the use of autologous cells and tissue culture on a scaffold as a mechanical support, which can enable the development of a valve substitute with remodeling capacity [6]. Several materials have been explored as potential scaffolds for heart valve tissue engineering, including natural and synthetic polymers, decellularized valvular platforms, 3D bioprinting, and hydrogel-based scaffolds, all of which are generating growing interest in the field [76,77]. To address the challenges associated with using autologous tissue-engineered heart valves (TEHV), in situ tissue engineering has been developed using an off-the-shelf implant that is designed to promote host cell adhesion and tissue formation, allowing the recipient’s body to regenerate and remodel the implant by exploiting the endogenous reparative capacity of the adult heart [78]. In fact, a multicenter clinical trial evaluating the safety and efficacy of the Cormatrix Cor TRICUSPID Valve is ongoing (NCT02397668) and currently recruiting patients aged 1–70 years. Preliminary results have been presented in 2020 and showed promising outcomes, with good valve performance. According to preclinical studies and off-label clinical cases, this tissue-engineered valve, by remodeling and ingrowth of native cells, can establish a connection between the annulus and papillary muscles, thus preserving normal geometry and function of the right ventricle, and immediately performs with low gradients after implantation [79].

The use of stem cell therapy, which has the ability to generate new cells of various tissues, has emerged as a promising and appealing treatment for heart failure and heart disease (Figure 1) [80]. Recent research has shown that valve endothelial-like cells, which exhibit similar morphology, molecular characteristics, and functions as primary VECs obtained from normal human aortic valves, can be efficiently derived from human pluripotent stem cells (hPSCs). Additionally, when these hPSC-derived valvular cells were placed on a de-cellularized porcine aortic valve matrix scaffold, they displayed greater proliferative and clonogenic potential than primary VECs. This has led to speculation that hPSC-derived valvular cells have the potential to be used in the production of next-generation tissue-engineered heart valves or valve organoids [81,82]. It is envisioned that autologous hPSC-derived valvular cells, from hPSCs obtained from the same patient in need of therapy, could further improve the performance and safety of the tissue-engineered heart valves (TEHV). To be sure, important biological issues, such as the impact of aging and senescence on patient donors, still limit the future application of such a revolutionizing strategy [2,83,84]. The upcoming clinical trials will gather crucial safety and performance data for next-generation heart valves, providing the foundation for their widespread clinical use. Additionally, they will offer valuable insights into the process of valve remodeling in humans, necessary to allow the development of valves with durable performance and native-like tissue configurations [83].

## 7. Conclusions

TV disease, and mainly TR, have received growing interest in the last years, although they are still undertreated compared to other valve diseases. There is a current need to improve the ability to risk stratify TR patients in order to obtain an early detection of valvulopathy and TR-related RV cardiomyopathy while also predicting valve dysfunction and/or response to TR treatment. Among the knowledge gaps in evidence, it is recognized that scientific efforts are still needed to eventually decipher the complete picture describing the etiopathogenesis of TV and TV-associated cardiomyopathy, and future advances to this aim may be achieved by combining emerging diagnostic imaging modalities with molecular and cellular studies. Surmounting the knowledge gap in TV disease would transform the treatment of congenital, as well as acquired, TV-related heart disease, and likewise would enable the development of personalized, in vitro cardiac valve disease models. However, despite the evolution of technical repair and replacement intervention for dysfunctional valves, none of the available prostheses is able to specifically face the challenges of replacement treatment: the degenerative process of heart valve prosthesis, with subsequent need for reoperation, and the incapacity to adapt to growth in young patients. Hopefully, the development of TEHV with repair, remodeling, and regeneration capacity will solve these clinical issues. Ideally, these valves would become available also for percutaneous implantation, avoiding the need for open chest surgery. Overall, molecular and cellular studies demonstrating new gene mutations, specific signal pathways alterations, hemodynamic influences, circulating biomarkers modifications, endothelial/valve progenitor cell impairment, and immune/inflammatory response, could better stratify tricuspid valvulopathy progression, identify timing for effective TV repair/replacement, and predict the usefulness of such treatment.

## Data Availability

Data sharing not applicable.
